# A Study of 2D Roughness Periodical Profiles on a Flat Surface Generated by Milling with a Ball Nose End Mill

**DOI:** 10.3390/ma17061425

**Published:** 2024-03-20

**Authors:** Mihaita Horodinca, Florin Chifan, Emilian Paduraru, Catalin Gabriel Dumitras, Adriana Munteanu, Dragos-Florin Chitariu

**Affiliations:** Digital Manufacturing Systems Department, Gheorghe Asachi Technical University of Iasi, 700050 Iasi, Romania; florin.chifan@academic.tuiasi.ro (F.C.); emilian.paduraru@academic.tuiasi.ro (E.P.); catalin-gabriel.dumitras@academic.tuiasi.ro (C.G.D.); adriana.munteanu@academic.tuiasi.ro (A.M.); dragos-florin.chitariu@academic.tuiasi.ro (D.-F.C.)

**Keywords:** 2D roughness profiles, milling, ball nose end mill, measurement, characterisation, curve (signal) fitting, fast Fourier transform

## Abstract

This paper presents a study of 2D roughness profiles on a flat surface generated on a steel workpiece by ball nose end milling with linear equidistant tool paths (pick-intervals). The exploration of the milled surface with a surface roughness tester (on the pick and feed directions) produces 2D roughness profiles that usually have periodic evolutions. These evolutions can be considered as time-dependent signals, which can be described as a sum of sinusoidal components (the wavelength of each component is considered as a period). In order to obtain a good approximate description of these sinusoidal components, two suitable signal processing techniques are used in this work: the first technique provides a direct mathematical (analytical) description and is based on computer-aided curve (signal) fitting (more accurate); the second technique (synthetic, less accurate, providing an indirect and incomplete description) is based on the spectrum generated by fast Fourier transform. This study can be seen as a way to better understand the interaction between the tool and the workpiece or to achieve a mathematical characterisation of the machined surface microgeometry in terms of roughness (e.g., its description as a collection of closely spaced 2D roughness profiles) and to characterise the workpiece material in terms of machinability by cutting.

## 1. Introduction

The surface roughness of steel work pieces machined by milling with ball nose cutters appears to be closely related to the interaction between the tool and the workpiece, and the machinability of the workpiece material by cutting. It depends mainly on the shape, geometry, and position of the tool (tilt angle, axial depth of cut, effective cutting diameter), the machining parameters (cutting speed, feed, and direction), the milling strategy (tool path pattern, step over distance), and the cutting forces (involved in the elastic deformations of the tool). Some non-systematic phenomena are also occasionally involved in the definition of this roughness: relative vibrations between tool and workpiece, self-excited vibrations, local variations in the hardness of the workpiece material, tool wear, cutting edge adhesion or fractures, etc. Therefore, under the most suitable milling conditions, the roughness is mainly characterised by a micro-geometry with a regular (periodic) shape, with equidistant pick (path) and feed interval scallops [1] on the pick and feed directions.

A better understanding of the interaction between tool and workpiece during any cutting (machining) process requires a thorough investigation of the surface roughness. The first approach to this investigation is the experimental sampling of the surface roughness description using appropriate equipment. The most common method to achieve this sampling is the use of contact profilometers [2,3,4,5,6,7,8,9,10] as a reliable but time-consuming method. Some other methods use the non-contact surface exploration by lasers [11], laser interferometry [12,13], laser confocal microscopes [14], optical systems [15,16,17,18], machine vision systems [19], or are inspired by research into the optical properties of surfaces (ability to split white light, diffractive properties) using scanning electron microscopy and atomic force microscopy [20].

As the description of a 3D surface roughness by sampling is generally obtained by joining many 2D roughness profiles (e.g., as a grid on pick and feed directions), the study of these surfaces mainly means a study of each of these 2D roughness profiles (2DRPs), often as a periodic evolution [2,4,5,8,11,15,16,18,20,21]. Some investigation techniques on this topic are available in the literature, most of which reveal the presence of numerous permanent sinusoidal components within these 2DRPs (as wavinesses [21], with dominants and some harmonics). Some previous studies indicate the availability of techniques to describe the components using synthetic rather than analytical methods, treating the 2DRPs mainly as digital time-dependent signals. The simplest synthetic description of the components can be obtained by digital filtering [22], in particular by selective band-pass filtering [8]. A relatively better approach to this synthetic description is possible using the power spectral density (by Fast Fourier Transform, FFT) of 2DRPs as time-dependent signals [2,7,8,19,21]. On an FFT spectrum (with amplitude on the *y*-axis and conventional frequency as the inverse of the wavelength on the *x*-axis), each significant sinusoidal component within a 2DRP is described as a peak. However, the availability of the FFT is generally seriously limited by the insufficiently low resolution of the conventional frequency (*R_cf_*) on the spectrum. The use of a high sampling rate (or sampling frequency *f_s_*) for 2DRP description (in order to have a high Nyquist limit *f_Nq_* = *f_s_*/2) should be mandatorily accompanied by a high number (*N*) of samples (or a large size length of 2DRP) in order to have a conveniently small resolution of conventional frequency *R_cf_* = *f_s_*/*N*. If this resolution is not small enough, some peaks in the spectrum will be missing or will have incorrectly described amplitudes (smaller than normal). This is a major inconvenience of the FFT that has not yet been resolved in these previous approaches. However, there is an additional drawback to the FFT analysis: the synthetic description of the sinusoidal components is incomplete (their phases at the origin of time are missing).

In some cases, the 2DRPs, considered as time-dependent signals, contain short sinusoidal components that do not persist permanently. For these situations (not considered in our work), where generally short oscillations (waves) occur transiently, the FFT analysis is not at all appropriate, but there are available other specific investigation techniques (inspired by the study of vibrations), e.g., based on Wavelet Transform (as Continuous Wavelet Transform [7], Frequency Normalised Wavelet Transform [23,24], and Wavelet Packet Transform [25]).

The main purpose of this work is focused on the study of periodic 2DRPs (considered as time-dependent signals) in order to determine the best analytical approximation of them (as a pattern), as close as possible to experimental evolutions, as a sum of significant sinusoidal components. Each sinusoidal component (analytically defined by the amplitude, a conventional angular frequency, and a phase at the conventional time origin) is a description of waviness on the machined surface of the workpiece. The inverse of the conventional frequency (as the conventional period) is the wavelength of the waviness.

Specifically, these 2DRPs are experimentally sampled in feed and pick directions (using a contact profilometer) on a theoretically flat surface milled with a ball nose end mill (on a steel workpiece in our approach). In order to analyse the 2DRP, a curve fitting procedure in Matlab R2019b (based on the Curve Fitting Toolbox) is favoured in this approach. In contrast to the FFT procedure (also discussed here), the curve fitting procedure can now be applied to relatively small (in length) 2DRPs, providing a high degree of accuracy in the analytical description of sinusoidal components. Similar to the FFT procedure, the curve fitting procedure has the same Nyquist limit (*f_Nq_* = *f_s_*/2); in other words, it is not possible to find out the analytical descriptions of sinusoidal components having conventional frequency above the Nyquist limit *f_Nq_*. The curve fitting procedure allows for an interesting approach: a 2DRP in the analytical description can be artificially resized by mathematical extrapolation (increasing the number of samples *N*, while keeping the same sampling rate *f_s_*). The accuracy of the FFT spectrum of this resized 2DRP is significantly improved due to a lower conventional frequency resolution, so that the FFT spectrum is now better suited to synthetically describe the content (in sinusoidal components) of a 2DRP. 

There is an interesting option in the 2DRP analysis: one period of the synthetically described roughness pattern is obtained by a special kind of moving averaging. This averaging drastically reduces both the sinusoidal components harmonically uncorrelated and the noise. The analytical description of this pattern is also achieved by curve fitting.

The following sections of this paper are organised as follows: Section 2 presents the materials and methods, Section 3 presents the results and discussions, and Section 4 presents the conclusions.

## 2. Materials and Methods

**Figure 1:**
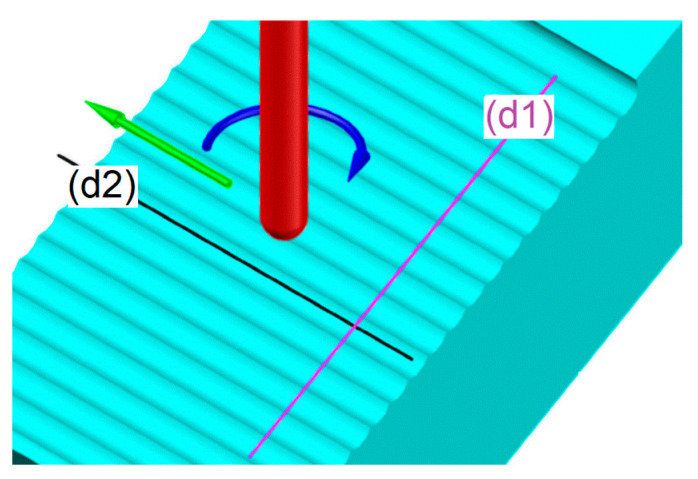
A conceptual description of the cutting process.

**Figure 2:**
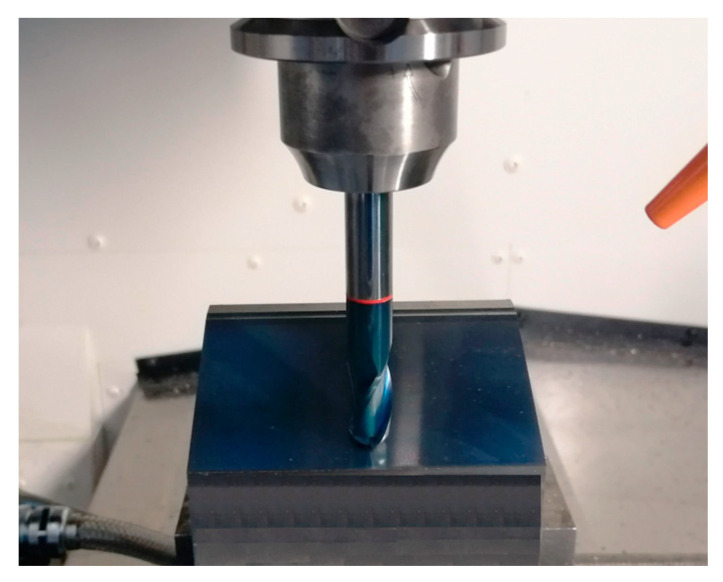
A view of the milling setup.

A flat surface was milled on a workpiece made of 90MnCrV8 steel (hardness 60 HRC) using a 12 mm diameter, 3 flute, TiAlN coated carbide ball nose end mill (as GARANT Diabolo solid carbide ball nose slot drill HPC 12 mm, from the Hoffmann Group, Bucharest, Romania), tilted at 25 degrees to the pick direction and perpendicular to the feed direction, with the following cutting regime parameters: 5200 rpm, 1560 mm/min feed rate, constant axial cutting depth of 0.1 mm and 0.4 mm step over (with theoretically equal pick-interval scallops height [26]). Figure 1 shows a conceptual description of the down milling process (with the workpiece in cyan, the tool in red, the feed direction in green, and the direction of rotation in blue). The magenta-coloured straight line (d1) represents the pick direction, and the black straight line (d2) represents the feed direction, with both conventionally used for experimental sampling of 2DRP. Figure 2 shows a view of the tool and workpiece (with the cutting process stopped) on an OKUMA GENOS M460R-VE CNC vertical machining centre (Charlotte, NC, USA).

**Figure 3:**
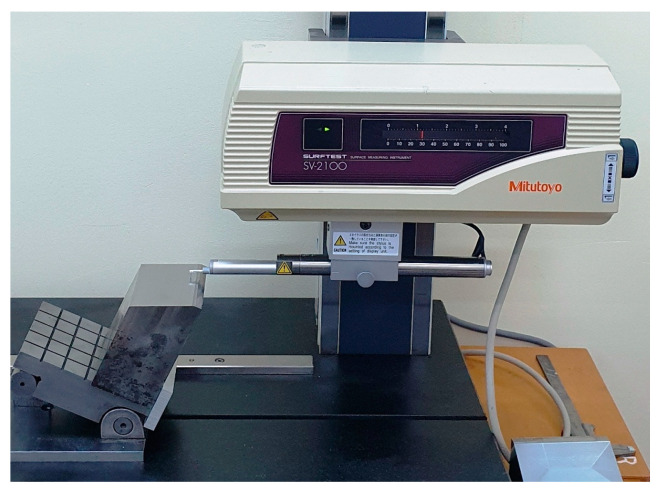
A view on the roughness sampling setup.

Figure 3 shows a view of the roughness sampling setup (using a SURFTEST SV-2100W4 contact profilometer, from Mitutoyo (Bucharest, Romania), with 0.0001 μm resolution, 2 μm stylus tip radius), with the flat milled surface placed in a horizontal position (here for sampling in pick direction).

**Figure 4:**
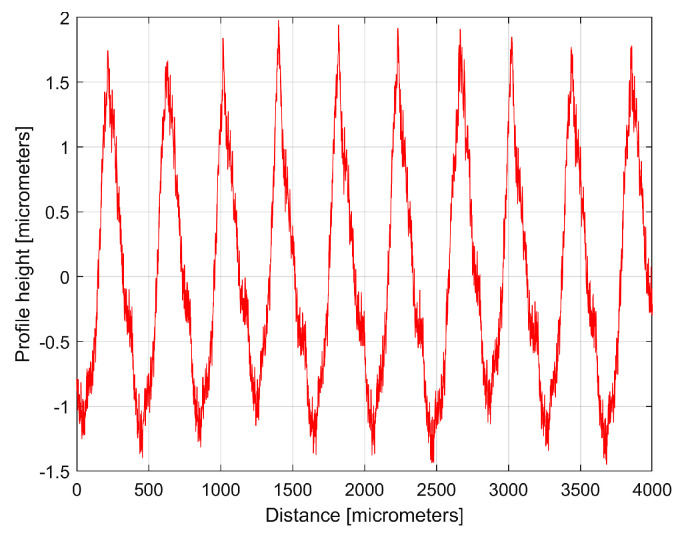
Graphical description of a 2DRP sampled on work piece, in the pick direction.

The numerical description of a 2DRP is delivered as a two-column .txt file describing *N* = 8000 equidistant samples (Δ*x* = 0.5 μm sampling interval between samples on the *x*-axis, for a total distance of 4 mm). This file can be easily loaded into Matlab R2019b and analysed as a time-dependent signal (by FFT and curve (signal) fitting). Figure 4 shows a 4 mm long 2DRP, sampled in the pick direction (plotted in Matlab).

Here the profilometer resolution (0.0001 μm) was experimentally confirmed (as the minimum describable variation of the *y*-coordinate). As expected, there is a dominant periodic component within the 2DRP of Figure 4. A rough estimation indicates that this dominant has 10 periods, with each period being equal to the milling step over (400 μm), and an average pick-interval scallop height of 2.5 μm.

**Figure 5:**
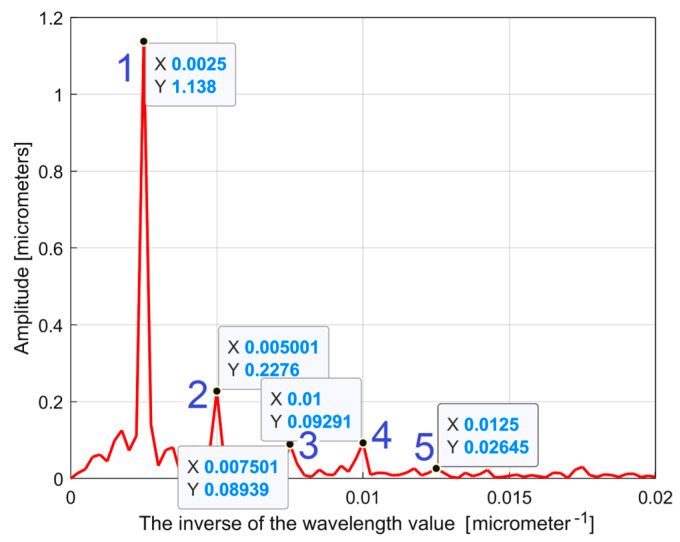
A partial view on the FFT spectrum of 2DRP from Figure 4.

Figure 5 shows a partial view of the FFT spectrum of this 2DRP with real amplitudes (in Matlab). The 2DRP from Figure 4 was processed with FFT as a time-dependent signal (the *x*-coordinates of the samples are seen as signal samples time; the *y*-coordinates are seen as signal level). The sampling interval Δ*x* on the *x*-axis (Δ*x* = 0.5 μm) is seen as the conventional sampling period Δ*t* on the *t*-axis. An *x*-coordinate on the *x*-axis of Figure 5 is equivalent to a conventional frequency or the inverse of a conventional period, or the inverse of a wavelength *λ*. A peak on the FFT spectrum (e.g., the highest peak 1, represented by an *x*-coordinate of 0.0025 μm^−1^ and a *y*-coordinate of 1.138 μm) indicates that there is a dominant sinusoidal component in the 2DRP with wavelength *λ* = 1/*x* (e.g., *λ*_1_ = 1/0.0025 = 400 μm for peak 1). This is exactly the step over value (pick feed) previously highlighted. In Figure 5, some other relevant peaks (2, 3, 4, and 5) represent sinusoidal components, harmonically correlated with the dominant, having the wavelengths *λ*_1_/2 = 200 μm, *λ*_1_/3 = 133.(3) μm, *λ*_1_/4 = 100 μm, and *λ*_1_/5 = 80 μm. The conventional sampling period Δ*t* = 0.5 μm corresponds to the sampling frequency (rate) *fs* = 1/Δ*t* = 2 μm^−1^ which is a conventional Nyquist limit (frequency) of *f_Nq_* = *f_s_*/2 = 1 μm^−1^. In other words, the smaller synthetically describable wavelength of a sinusoidal component within the 2DRP by FFT spectrum is defined as *λ_min_* = (*f_Nq_*)^−1^ = (*f_s_*/2)^−1^ = 1 μm.

However, as Figure 5 clearly shows, the conventional frequency resolution *R_cf_* = *f_s_*/*N* = 2/8000 = 0.00025 μm^−1^ is not small enough in order to describe an accurate spectrum. In the spectrum from Figure 5 there are only 0.02/*R_cf_* = 0.02/0.00025 = 80 samples. There are certainly other harmonics (with higher conventional frequencies) that are not visible in the spectrum. A longer 2DRP (obtained by increasing the number of samples at the same sampling frequency) significantly reduces the conventional frequency resolution. It should also be noted that the FFT spectrum does not provide the phase at the origin of the conventional time (*x* = 0) for sinusoidal components. A better approach proposed in this paper considers that within the *y*(*x*) 2DRP there is a consistent deterministic part *y_d_*(*x*) and a less significant non-deterministic part *y_nd_*(*x*), mainly as noise, with *y*(*x*) = *y_d_*(*x*) + *y_nd_*(*x*). In general, for periodic 2DRPs, this deterministic part *y_d_*(*x*) can be described as the sum of *n* sinusoidal components:

(1) $$y_{d}\left(x\right)=\sum_{j=1}^{n}y_{dj}\left(x\right)=\sum_{j=1}^{n}A_{j}\cdot \sin\left(\omega_{j}\cdot x+\varphi_{j}\right)\;$$

In Equation (1), *A_j_* are amplitudes, *ω_j_* are conventional angular frequencies (related by wavelengths *λ_j_*, with *ω_j_* = 2*π*/*λ_j_*), and *φ_j_* are conventional phase shifts at the origin (*x* = 0). Here, *x* (the current position of the profilometer stylus on the *x*-axis) plays the role of time.

**Figure 6:**
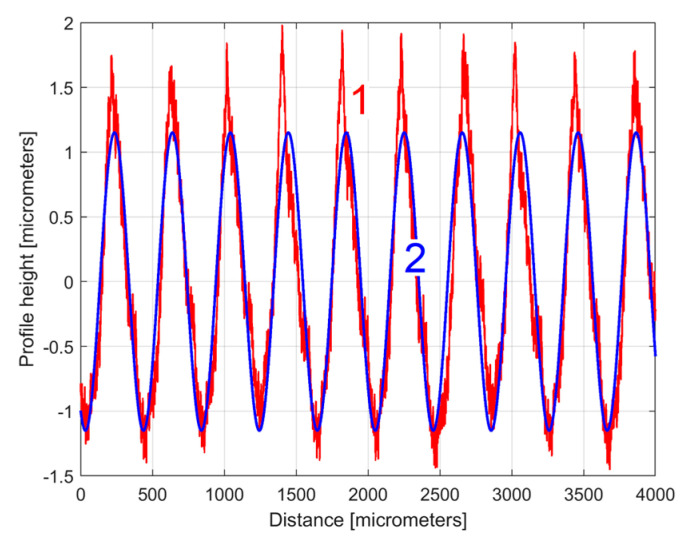
1—The 2DRP from Figure 4; 2—The first (dominant) sinusoidal component (*y_d_*_1_(*x*)) found by curve (signal) fitting (Equation (2)).

The curve (signal) fitting procedure (using the Curve Fitting Tool from Matlab) allows for the values of *A_j_*, *ω_j_*, and *φ_j_* to be determined with a good approximation. A sine model (as f(x) = a1*sin(b1*x + c1)) was used for a first fit, with *x*—coordinates as X data and *y*—coordinates as Y data. In this model, a1, b1, and c1 play the role of *A*_1_, *ω*_1_, and *φ*_1_ values in defining the first sinusoidal component *y_d_*_1_(*x*). The first curve fit gives *A*_1_ = 1.151 μm, *ω*_1_ = 0.01558 rad/μm, and *φ*_1_ = 4.1871 rad, whereby typically this curve fitting procedure finds the description of the highest amplitude component. It systematically searches for those suitable *A*_1_, *ω*_1_, and *φ*_1_ values that satisfy the condition: $\sum \left\lfloor y\left(x\right)-y_{d1}\left(x\right)\right\rfloor =min$. This first sinusoidal component *y_d_*_1_(*x*) is shown (as dominant) in blue in Figure 6, superimposed on *y*(*x*), shown in red (an evolution already described in Figure 4). The component *y_d_*_1_(*x*) can be described mathematically as:

(2) $$y_{d1}\left(x\right)=A_{1}\cdot \sin\left(\omega_{1}\cdot x+\varphi_{1}\right)=1.151\cdot \sin\left(0.01557\cdot x+4.1871\;\right)$$

**Figure 7:**
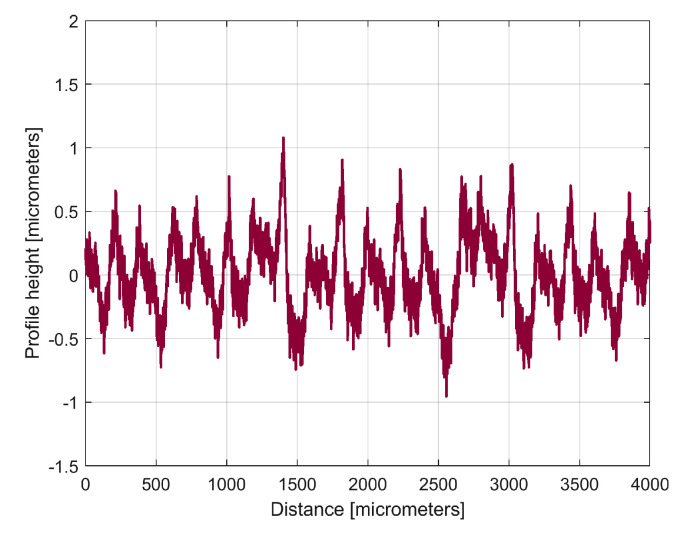
The first residual 2DRP after first analysis by curve fitting (as *r*_1_(*x*) = *y*(*x*) *− y_d_*_1_(*x*)).

The description of *y_d_*_1_(*x*) from Equation (2) allows for the mathematical removal from *y*(*x*), with the result shown in Figure 7 as the first residual (*r*_1_(*x*)) of 2DRP, as *r*_1_(*x*) = *y*(*x*) − *y_d_*_1_(*x*), after the first curve fitting (drawn at the same scale as Figure 6). The decrease in the *y*-coordinates of the residual profile is additional evidence of the quality of the mathematical description of *y_d_*_1_(*x*) found by curve fitting.

It is clear that the dominant component *y_d_*_1_(*x*) does indeed fit *y*(*x*). Its amplitude *A*_1_ is close to that shown in the FFT spectrum (peak 1), and its wavelength *λ*_1_ = 2*π*/*ω*_1_ = 2π/0.01558 = 403.285 μm is close to the step over value or pick feed (400 μm) during the milling process. The conventional frequency of *y_d_*_1_(*x*) is 1/*λ*_1_ = 0.002479 μm^−1^, which is more precisely described by comparison with Figure 5, as related to the first peak (there 1/*λ*_1_ = 0.0025 μm). Related by the difference between the measured wavelength *λ*_1_ = 403.285 μm (determined by curve fitting) and the pick feed (400 μm, as theoretical wavelength *λ*_1_ generated by the CNC machining centre), a logical conclusion must be drawn: we suspect an inaccurate control of the *x* movement of the contact profilometer during the 2DRP measurement (involved in the measured *λ*_1_) rather than inaccurate control of the pick feed during the milling process. In Figure 6, there are not exactly ten periods of the dominant *y_d_*_1_(*x*), as the ratio between the 2DRP length (4000 μm) and the theoretical wavelength (400 μm) suggests.

It is clear that this procedure can be repeated many times in an identical way (automatically, by programming in Matlab), and the mathematical description of the sinusoidal component *y_dj_*(*x*) can be found by curve fitting of the (*j −* 1)th residual of 2DRP, as *r_j−_*_1_(*x*), described by Equation (3):

(3) $$r_{j-1}\left(x\right)=y\left(x\right)-\sum_{k=1}^{j-1}y_{dk}\left(x\right)\;$$

Of course, in the curve-fitting procedure (as in the case of the FFT spectrum), exceeding of the Nyquist limit is forbidden (*ωj <* 2*πf_Nq_* or *λ_j_ >* (*f_Nq_*)*^−^*^1^). 

Hypothetically, considering that *y_nd_*(*x*) = 0, a perfect mathematical description of *y_d_*(*x*) (after *n* similar curve-fitting steps), should produce an *r_n_*(*x*) = 0 for the *n*th residual of 2DRP (graphically represented as a straight line placed exactly on the *x*-axis).

The viability of this method of determining the mathematical description of a roughness profile (using a similar curve-fitting method developed in Matlab) has previously been demonstrated [27] in the analysis of other types of complex signals (vibration, active electrical power, instantaneous angular speed, etc.) containing many sinusoidal components.

## 3. Results and Discussion

### 3.1. Analysis of 2D Roughness Profiles in the Pick Direction by Curve Fitting

**Figure 8:**
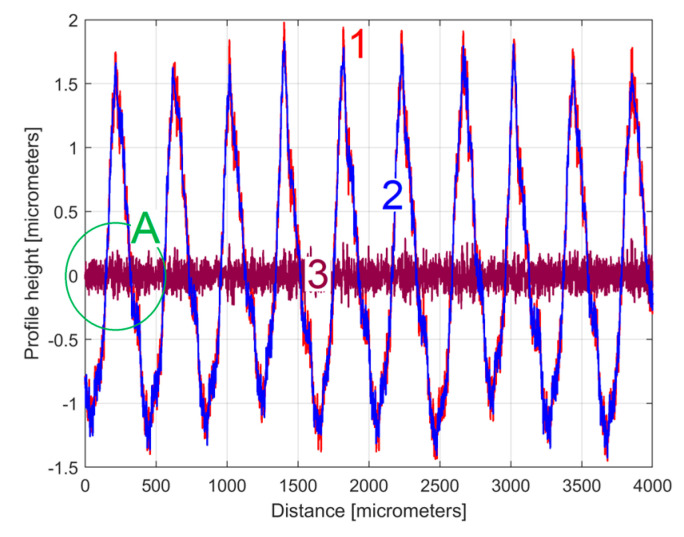
1—The 2DRP; 2—An approximation of *y_d_*(*x*) through *y_dh_*(*x*) with 122 components; 3—The 122nd residual *r*_122_(*x*).

**Figure 9:**
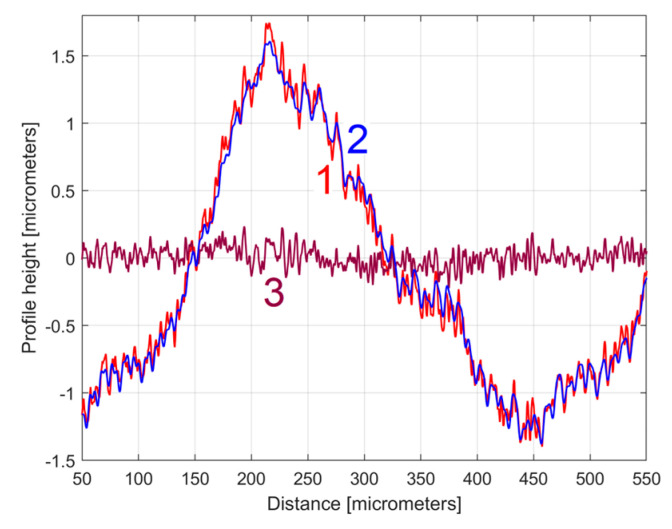
A zoom-in detail in area A from Figure 8.

The analysis of the previously sampled 2DRP (Figure 4) was similarly performed using repetitively this curve fitting procedure a further 121 times. The mathematical description of these 122 sinusoidal components within *y*(*x*) was found (these components having amplitudes greater than the resolution of the contact profilometer). Figure 8 shows the 2DRP (already shown in red in Figure 4 and Figure 6) superimposed on an approximation of *y_d_*(*x*) by mathematical addition of these 122 sinusoidal components (shown in blue). In the same figure, the 122nd residual of 2DRP (*r*_122_(*x*)), shown in purple, is superimposed. Figure 9 shows a zoomed section of area A from Figure 8.

It is obvious that there is a good fit between the approximation of *y_d_*(*x*) and *y*(*x*). Compared to Figure 7, there is a significantly smaller residual of 2DRP, which mainly describes the non-deterministic part *y_nd_*(*x*) of *y*(*x*) and the measurement noise. In a simple approach, this noise—which does not significantly affect the fitting results—can be greatly reduced by numerical low-pass filtering.

As is well known [28], any evolution of a signal in time (or similar, e.g., this 2DRP in pick direction) can be well approximated as a sum of sinusoidal components. In our approach, it is more interesting to find the approximate analytical description of 2DRP (strictly related to the milling process) as a sum of harmonically correlated sinusoidal components (as *y_dh_*(*x*)) with a fundamental at 0.01557 rad/μm as conventional angular frequency *ω*_1_ (Equation (2)) related by pick feed or step over and some harmonics (at 2·0.01557 rad/μm, 3·0.01557 rad/μm, etc.). In other words, the deterministic part of *y*(*x*) should be seen as *y_d_*(*x*) = *y_dh_*(*x*) + *y_dnh_*(*x*), where *y_dnh_*(*x*) is a sum of sinusoidal non-harmonically correlated components. Of course, this new type of approximation is available here because *y_dh_*(*x*) is dominant (*y_d_*(*x*) *≈ y_dh_*(*x*)).

Among the 122 identified sinusoidal components, 30 components (Hi) were found to be well harmonically correlated (and involved in the definition of *y_dh_*(*x*) from Equation (4)) with a good approximation, with the values of *A_Hi_* (amplitudes), *ω_Hi_* (conventional angular frequencies), and *φ_Hi_* (phases in origin) given in Table 1.

(4) $$y_{dh}\left(x\right)\approx \sum_{i=1}^{30}A_{Hi}\cdot \sin\left(\omega_{Hi}\cdot x+\varphi_{Hi}\right)$$

Some harmonics in Table 1 are missing (e.g., H6, H17, H20, H21, etc.).

**Figure 10:**
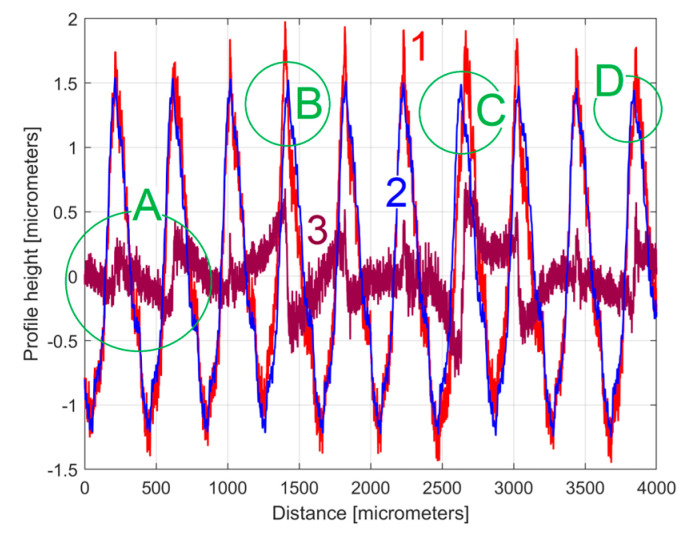
1—The 2DRP; 2—An approximation of *y_d_*(*x*) with the profile *y_dh_*(*x*) with 30 components described in Table 1; 3—The 30th residual *r*_30_(*x*).

**Figure 11:**
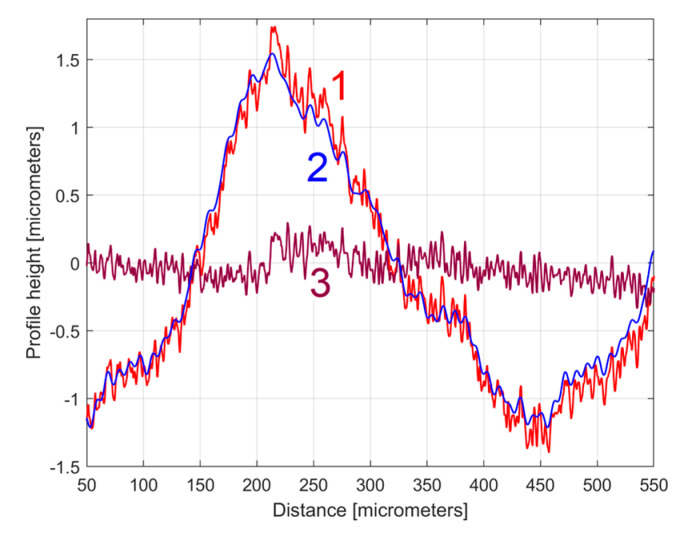
A zoom-in detail in area A from Figure 10.

Figure 10 shows an equivalent of Figure 8 but with an approximation of *y_d_*(*x*) by *y_dh_*(*x*), according to Equation (4) and Table 1. Figure 11 shows a zoomed detail in area A of Figure 10 (similar to Figure 9).

A comparison of Figure 10 and Figure 11 with Figure 8 and Figure 9 shows that the fit is acceptable but less good than before, an aspect that is well highlighted by the evolution of the residual (*r*_30_(*x*)). In particular, in some areas (e.g., B, C, and D in Figure 10) the fit between *y*(*x*) and *y_dh_*(*x*) is locally less good. There are several reasons for this mismatch. Firstly, we should consider the angular position of the milling tool (due to its rotation). This position was not necessarily the same each time when its axis intersects the line (e.g., (d1) on Figure 1) where the 2DRP was sampled (the pick-interval scallops geometry on this line from the working piece is slightly different). Secondly, there is a variable flexional deformation of the milling tool in the direction of this line (pick feed direction).

**Figure 12:**
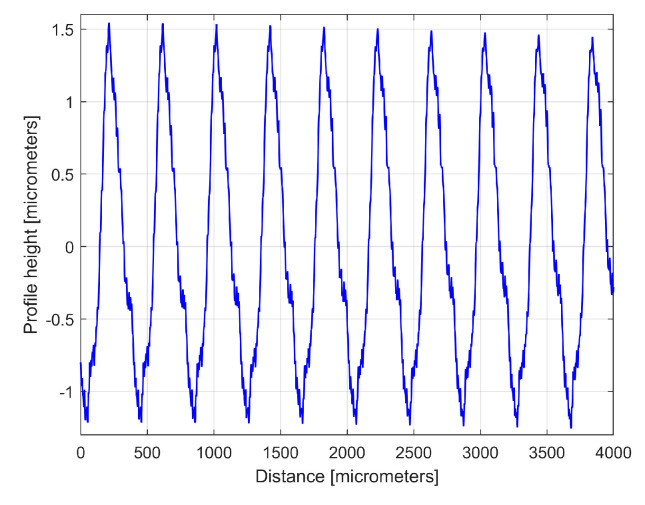
The evolution of the *y_dh_*(*x*) profile.

However, in this approach, the evolution of *y_dh_*(*x*) (shown separately in Figure 12) provides one of the best characterisations of the 2DRP, which is systematically related to the interaction between the milling tool and the workpiece (and obviously by the properties of its material).

Due to a small imprecision in the curve (signal) fitting process, there is not a perfect harmonic correlation between the 30 components within *y_dh_*(*x*), as clearly indicated in Table 1 (with *ω_Hi_ ≈ Hi·ω_H_*_1_ or *λ_Hi_ ≈ λ_H_*_1_/*Hi*), and the evolution of *y_dh_*(*x*) from Figure 12 is not strictly periodic, as expected.

This inconvenience can be easily avoided by roughly considering *ω_Hi_* = *H_i_·ω_H_*_1_ in Equation (4). A more rigorous approach is to replace above the conventional angular frequency *ω_H_*_1_ with a more precisely equivalent value *ω_He_*_1_, calculated as follows:

(5) $$\omega_{He1}={\left(\sum_{i=1}^{30}\frac{A_{Hi}}{A_{H1}}\right)}^{-1}\cdot \sum_{i=1}^{30}\frac{A_{Hi}}{A_{H1}}\;\frac{\omega_{Hi}}{H_{i}}=0.0155785\;rad/\mu m\;$$

In Equation (5), *ω_He_*_1_ is the weighting (by amplitude *A_Hi_*) of the conventional angular frequency *ω_Hi_*of each harmonic, relative to the amplitude *A_H_*_1_ of the first harmonic H1 (as dominant). However, here particularly, there is no significant difference between *ω_H_*_1_ and *ω_He_*_1_.

**Figure 13:**
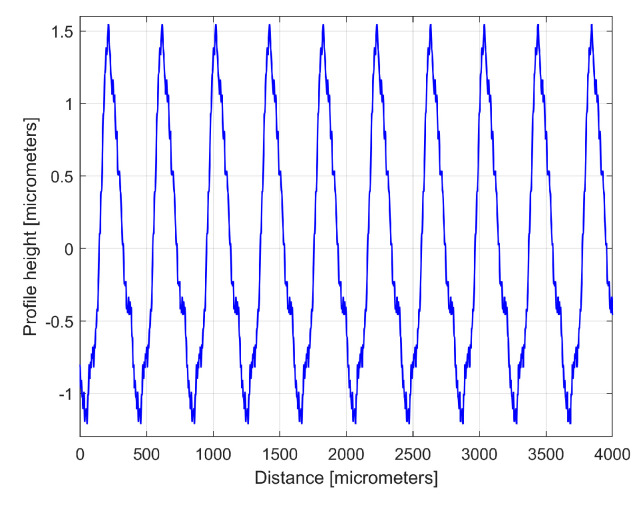
The evolution of the *y_dhe_*(*x*) profile.

With this value *ω_He_*_1_, the description of *y_dh_*(*x*) from Equation (4) can be rewritten as *y_dhe_*(*x*) according to Equation (6) and plotted according to Figure 13.

(6) $$y_{dhe}\left(x\right)=\sum_{i=1}^{30}A_{Hi}\cdot \sin\left(H_{i}{\cdot \omega }_{He1}\cdot x+\varphi_{Hi}\right)$$

**Figure 14:**
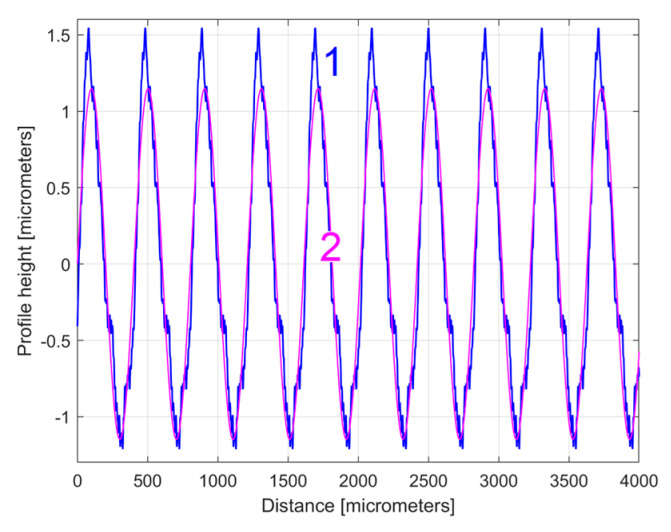
1—The evolution of *y_dhe_*_0_(*x*) profile; 2—The evolution of the dominant component H1_0_.

This *y_dhe_*(*x*) profile can be accepted as a systematic characterisation (pattern) of the 2DRP in the pick direction. An even more interesting characterisation is obtained if this *y_dhe_*(*x*) profile is described by artificially shifting the origin on *x*-axis (*x* = 0) in the abscissa (*2π* − *φ_H_*_1_)/*ω_He_*_1_ of the first zero crossing (from negative to positive values) of the dominant sinusoidal component (H1 in Table 1). Now the profile *y_dhe_*(*x*) becomes *y_dhe_*_0_(*x*), described mathematically by Equation (7) and shown graphically in blue in Figure 14. Here, the magenta curve describes the dominant component (H1), also shifted to new origin (as H1_0_), with *ω_H_*_1_ replaced by *ω_He_*_1_.

(7) $$y_{dhe0}\left(x\right)=\sum_{i=1}^{30}A_{Hi}\cdot \sin\left[H_{i}{\cdot \omega }_{He1}\cdot (x+\frac{2\pi -\varphi_{H1}}{\omega_{He1}})+\varphi_{Hi}\right]\;$$

With Equation (7) rewritten as Equation (8), this motion to a new origin is equivalent to a positive phase shift (with *H_i_·*(*2π − φ_H_*_1_)) at the origin for each sinusoidal component.

(8) $$y_{dhe0}\left(x\right)=\sum_{i=1}^{30}A_{Hi}\cdot \sin\left[H_{i}{\cdot \omega }_{He1}\cdot x+\varphi_{Hi}+H_{i}\cdot {(2\pi -\varphi }_{H1}))\right]$$

**Figure 15:**
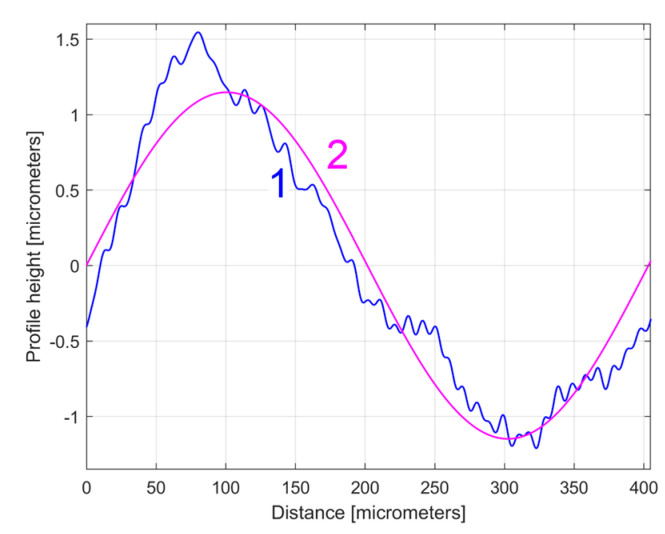
A detail of Figure 14 with the first period of the *y_dhe_*_0_(*x*) profile and H1_0_.

Figure 15 shows a zoomed detail of Figure 14, with a first period of the *y_dhe_*_0_(*x*) profile and of the H1_0_ sinusoidal component.

This *y_dhe_*_0_(*x*) type of 2DRP is useful when comparing two (or more) 2DRPs sampled under similar conditions. In this approach, a second 2DRP was sampled on the same flat milled surface on a straight line parallel to (d1) on the pick direction, with a randomly chosen distance between (several millimetres). 

**Figure 16:**
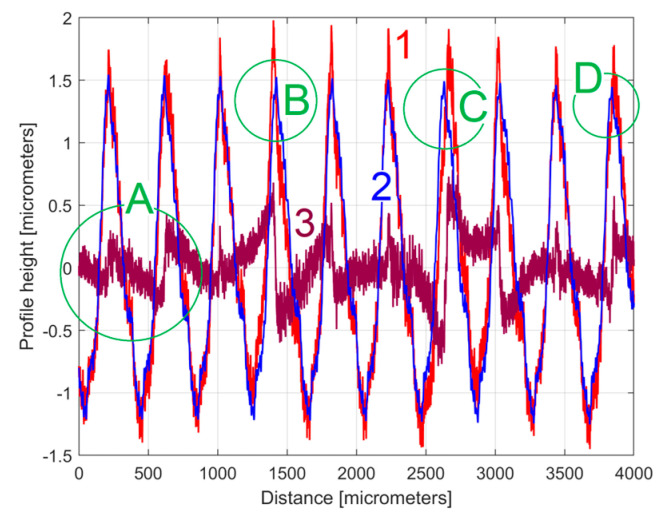
1—A new 2DRP; 2—An approximation of *y_d_*(*x*) with *y_dh_*(*x*) profile having 12 components; 3—The 12th residual *r*_12_(*x*).

As an equivalent to Figure 10, Figure 16 shows this new 2DRP (as *y*(*x*), in red) with the same number of samples (8000) and sampling interval (Δ*x* = 0.5 μm), overlaid with the *y_dh_*(*x*) profile (in blue) and the residual (in purple). This time only 12 harmonically related sinusoidal components inside *y_dh_*(*x*) were found (Table 2) among the 122 sinusoidal components in *y_d_*(*x*). The areas A–D mark some mismatches between the *y*(*x*) and *y_dh_*(*x*) profiles.

**Figure 17:**
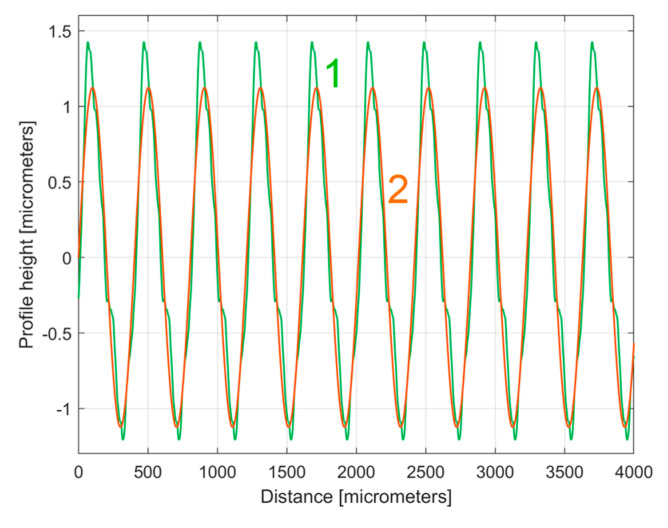
1—The evolution of the *y_dhe_*_0_(*x*) profile; 2—The evolution of the dominant component H1_0_. An equivalent of Figure 14.

As an equivalent to Figure 14, Figure 17 shows the evolution of the *y_dhe_*_0_(*x*) profile (in green) superimposed on the evolution of the dominant component H1_0_ (in brown).

**Figure 18:**
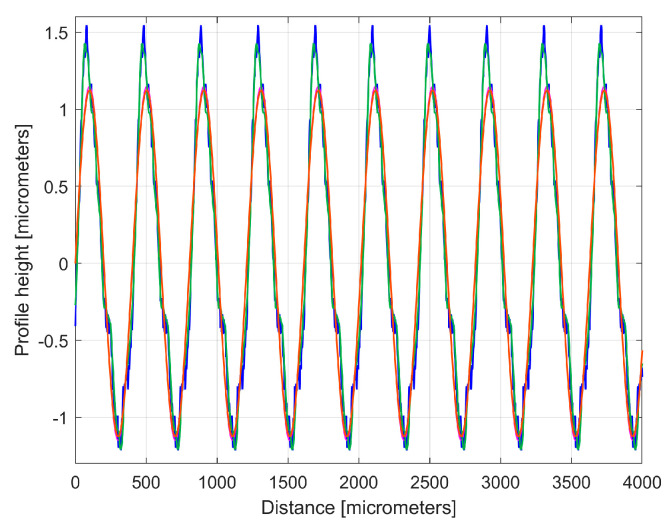
An overlap of both *y_dhe_*_0_(*x*) profiles (for 1st and 2nd 2DRP) and their dominants H1_0_.

**Figure 19:**
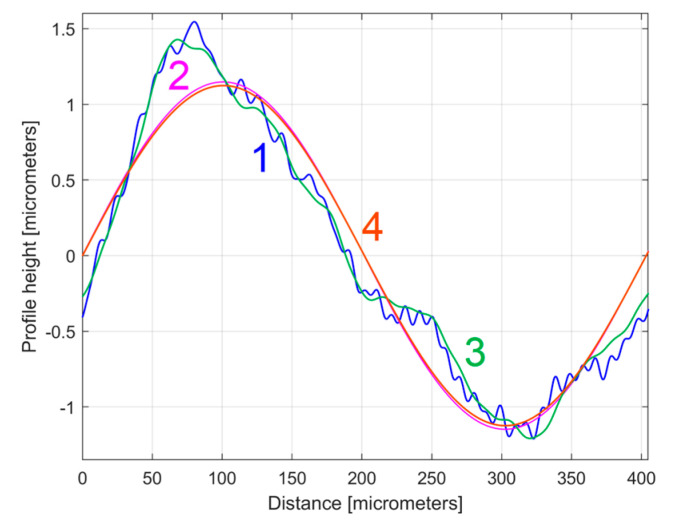
A zoomed detail of Figure 18: 1, 3—the *y_dhe_*_0_(*x*) profiles; 2, 4—the dominants H1_0_.

It is interesting here to note the similarities (by comparison) between the *y_dhe_*_0_(*x*) profiles (from Figure 14 and Figure 17) by their overlap in Figure 18. This is possible because both profiles start from a zero crossing (from negative to positive *y*-ordinates) of their dominant component H1_0_. A zoomed detail of the first period of Figure 18 is shown in Figure 19.

As can be seen in Figure 18 and especially in Figure 19, there are strong similarities between the *y_dhe_*_0_(*x*) profiles 1 and 3 (and also between the dominant components H1_0_ 2 and 4). This proves that the proposal of this *y_dhe_*_0_(*x*) pattern is a useful approach in a comparative analysis of 2DRPs sampled under similar conditions (especially direction) on a flat milled surface with a ball nose end mill. 

#### 3.1.1. Synthesis of a 2D Roughness Profile Pattern on a Period by Profile Averaging

There is another simple way of obtaining a synthetic (non-analytical) description of a pattern useful for characterizing the periodic 2DRPs graphically represented by *m* conventional periods: the *y*-coordinate of a point on this pattern (as *y_ap_*(*x*)) is an average of the *y*-coordinates of *m* samples of 2DRP (calculated using a moving average, with *m* samples selectively selected for averaging). The distance (measured on the *x*-axis) between each two consecutive samples considered within the average is exactly the conventional period of the dominant H1, as the equivalent wavelength *λ_He_*_1_ calculated with *λ_He_*_1_ = 2*π*/*ω_He_*_1_. A *y*-coordinate value of this—pattern *y_ap_*(*x*) is determined by calculation as Equation (9):

(9) $$y_{ap}\left(x\right)=\frac{1}{m}\sum_{i=0}^{m-1}y\left(x+i\cdot \lambda_{He1}\right)\;with\;x=0÷\;\lambda_{He1}\;$$

The length of this *y_ap_*(*x*) pattern is exactly the conventional period (the wavelength *λ_He_*_1_). A better approach is to describe this *y_ap_*(*x*) pattern starting from the zero crossing of the dominant H1 (as *y_ap_*_0_(*x*), Equation (10)), where this starting point still has the *x*-coordinate (*2π* − *φ_H_*_1_)/*ω_He_*_1_.

(10) $$y_{ap0}\left(x\right)=\frac{1}{m}\sum_{i=0}^{m-1}y\left(x+\frac{{2\pi -\varphi }_{H1}}{\omega_{He1}}+i\cdot \lambda_{He1}\right)\;with\;x=0÷\;\lambda_{He1}\;$$

Here, *x* is the *x*-coordinate of a generic point on the pattern *y_ap_*_0_(*x*). In Equation (10), in almost all previous equations (except Equation (5)) and in the sampled 2DRP, the *x*-coordinate is described numerically as *x* = *l·*Δ*x* for the *l*th sample, *l* = 1 ÷ *N*. Here above, *x* + (2*π − φ_H_*_1_)/*ω_He_*_1_+ *i·λ_He_*_1_ and *x* + *i·λ_He_*_1_ in Equation (9) are also *x*-coordinates (numerically described) of samples placed on 2DRP. 

**Figure 20:**
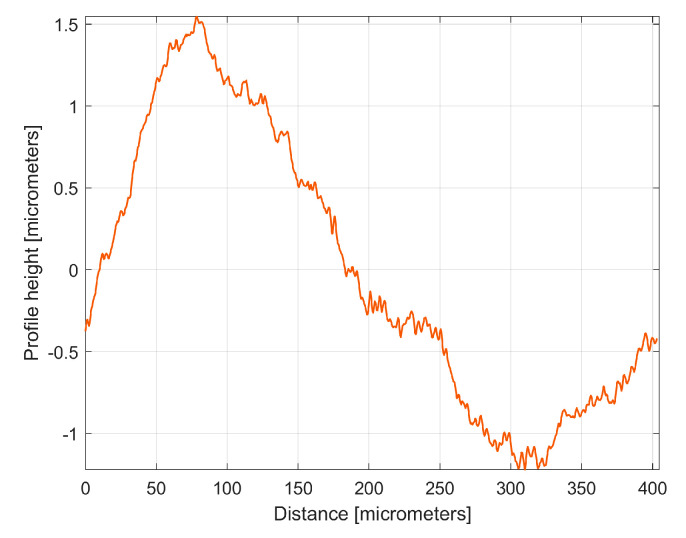
The *y_ap_*_0_(*x*) pattern of the first 2DRP.

**Figure 21:**
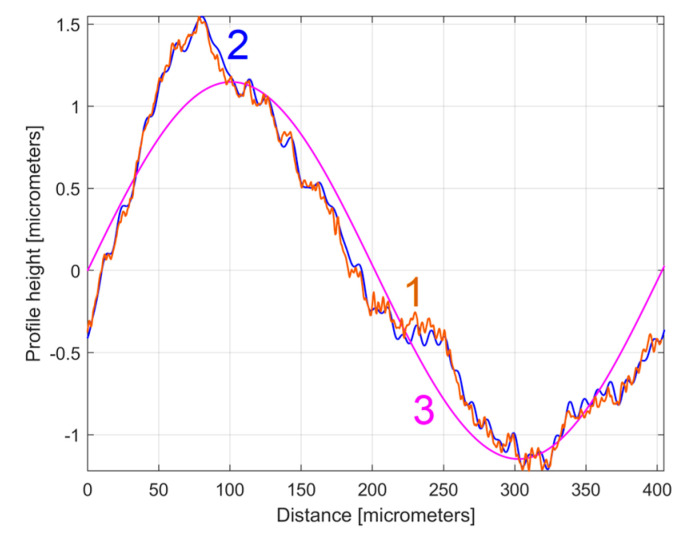
1—The *y_ap_*_0_(*x*) pattern of the first 2DRP; 2—The first period of the *y_dhe_*_0_(*x*) profile; 3—The dominant H1_0_.

Figure 20 shows this *y_ap_*_0_(*x*) pattern, established by averaging, for the first sampled 2DRP (with *m* = 9). This averaging (acting as a form of digital filtering) greatly attenuates the non-sinusoidal components (noise) as well as the harmonic uncorrelated components with the dominant H1, but it retains the harmonically correlated (averaged) components if they occur systematically. In other words, it is expected that this *y_ap_*_0_(*x*) pattern is similar with the first period of the *y_dhe_*_0_(*x*) profile (already shown in Figure 15). This is fully confirmed in Figure 21, where the *y_ap_*_0_(*x*) pattern, the *y_dhe_*_0_(*x*) profile, and the dominant H1_0_ (the first periods) are overlapped. 

**Figure 22:**
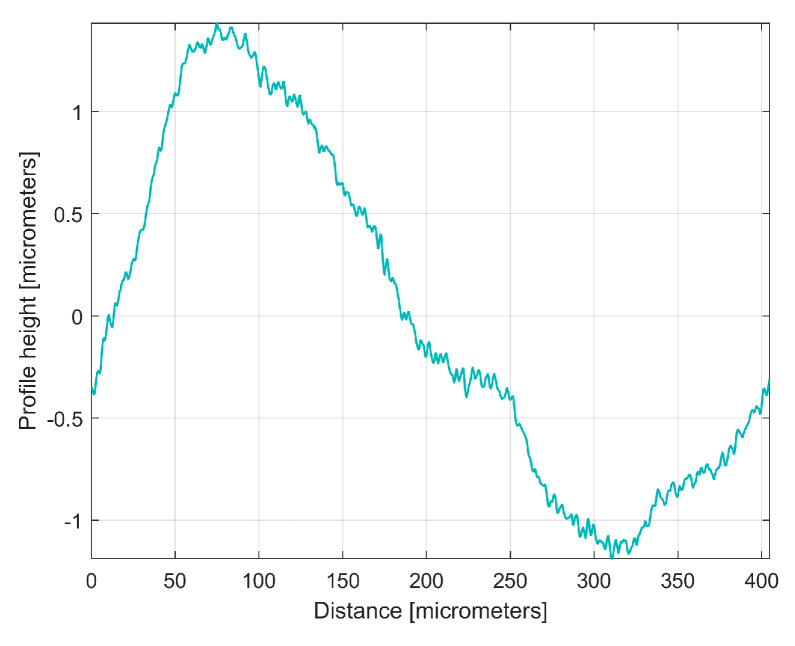
The *y_ap_*_0_(*x*) pattern of the 2nd 2DRP.

Similar considerations can be made for the *y_ap_*_0_(*x*) pattern of the 2nd 2DRP sampled in the pick direction, as shown in Figure 22.

**Figure 23:**
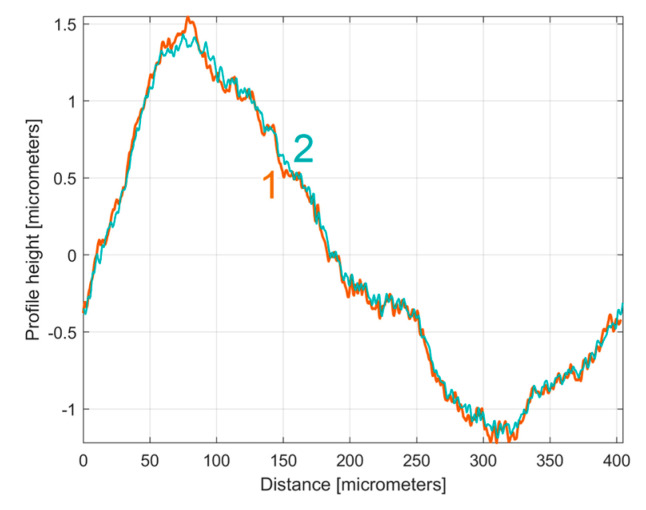
A graphical overlapping of the *y_ap_*_0_(*x*) patterns: 1—for 1st 2DRP; 2—for 2nd 2DRP.

There is an interesting utility of these *y_ap_*_0_(*x*) patterns, similar to the utility of the first periods of the *y_dhe_*_0_(*x*) profiles, already shown in Figure 19. It allows us to synthetically characterize the roughness profiles, possibly for comparison. As an example, Figure 23 shows the overlap of the *y_ap_*_0_(*x*) patterns for both sampled 2DRPs in the pick direction. 

As expected, there is a very good similarity between the *y_ap_*_0_(*x*) patterns, which is even better than between the *y_dhe_*_0_(*x*) profiles (from Figure 19).

**Figure 24:**
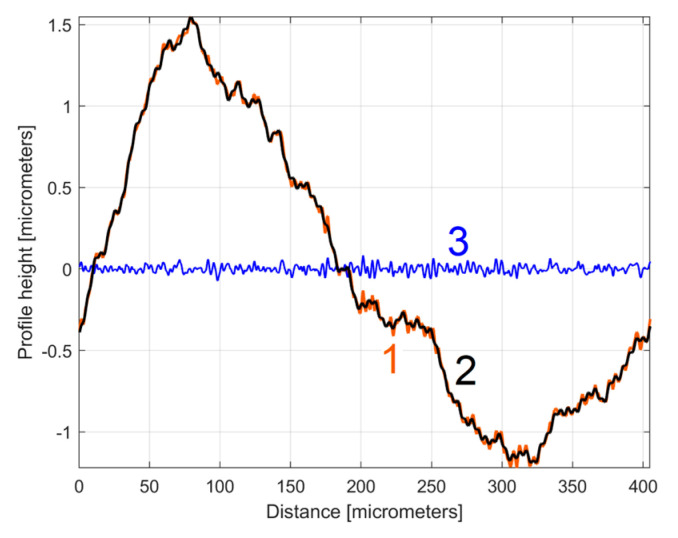
1—The *y_ap_*_0_(*x*) pattern for 1st 2DRP; 2—The first period from the *y_dhe_*_0*t*_(*x*) profile; 3—The residual *y_ap_*_0_(*x*) − *y_dhe_*_0*t*_(*x*).

There is an interesting and simpler way to obtain a more trustworthy mathematical description of the *y_dhe_*_0_(*x*) profiles (as *y_dhe_*_0*t*_(*x*)) through analysis by curve (signal) fitting of the mathematically extended *y_ap_*_0_(*x*) patterns over a large number of periods (e.g., 10), while keeping the same sampling interval Δ*x* = 0.5 μm. Figure 24 shows the *y_ap_*_0_(*x*) pattern for the 1st 2DRP, the first period of the *y_dhe_*_0*t*_(*x*) profile, and the residual *y_ap_*_0_(*x*) − *y_dhe_*_0*t*_(*x*). The *y_dhe_*_0*t*_(*x*) profile is described as the sum of 30 harmonically correlated sinusoidal components found in the extended *y_ap_*_0_(*x*) pattern. Now, by comparison with the results from Figure 21, the similarity between the *y_ap_*_0_(*x*) pattern and the *y_dhe_*_0*t*_(*x*) profile is consistently improved.

**Figure 25:**
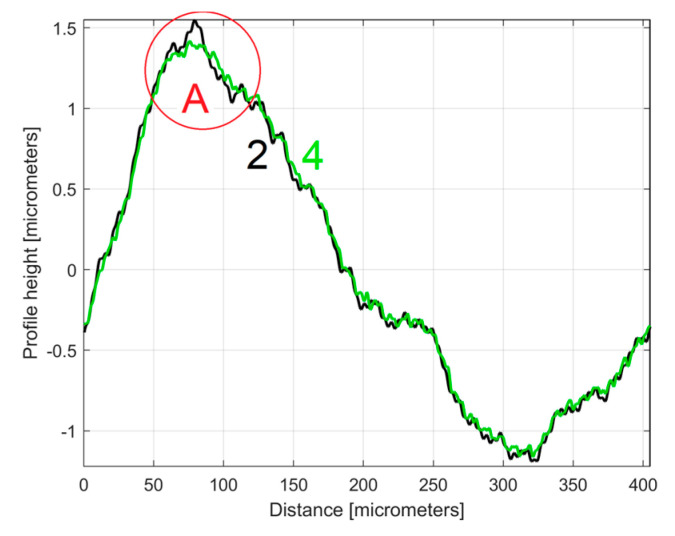
The first period of the *y_dhe_*_0*t*_(*x*) profiles: 2—for 1st 2DRP; 4—for 2nd 2DRP.

The first periods from these *y_dhe_*_0*t*_(*x*) profiles (for each 2DRP, each one as a sum of 30 harmonically correlated sinusoidal components) obtained using this new approach are shown in Figure 25. 

Compared to Figure 19 (where the profiles *y_dhe_*_0_(*x*) are overlapped), in Figure 25 the *y_dhe_*_0*t*_(*x*) profiles are much more similar, with the exception of area A. As expected, there are very strong similarities between the *y_dhe_*_0*t*_(*x*) profiles of Figure 25 and the *y_ap_*_0_(*x*) patterns of Figure 23.

#### 3.1.2. An Approach on FFT Spectrum in 2D Roughness Profile Description

There is another interesting resource that can be exploited related to the mathematical description of the *y_dh_*(*x*) profile, and in particular the *y_dhe_*(*x*) profile. As already mentioned in Section 1, the length of any of two analytical profiles can be artificially increased by mathematical extrapolation (by increasing the number of samples from *N* to *p·N*), while keeping the same sampling rate *f_s_* (or the same sampling interval Δ*x* = 0.5 μm). In this way, the conventional frequency resolution (as *R_cfe_*) of the FFT spectrum for each of the two extrapolated profiles (*R_cfe_* = *f_s_*/*pN*) is significantly reduced (by *p* times compared to the spectra of the original profiles having *R_cf_* = *f_s_*/*N* conventional frequency resolution), while the Nyquist limit remains unchanged. The quality description of the sinusoidal profile components by means of the FFT spectrum increases significantly. 

**Figure 26:**
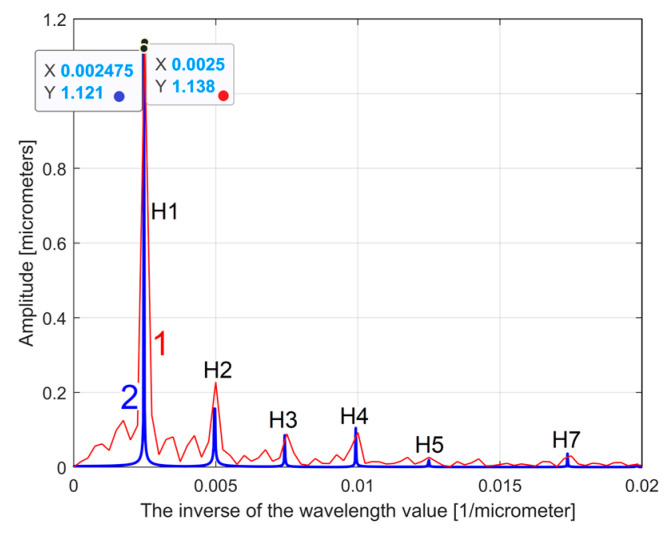
A partial view of the FFT spectrum: 1—of the *y*(*x*) profile; 2—of the extrapolated *y_dhe_*(*x*) profile, with *p* = 10. The peaks H1–H7 depict harmonic correlated components.

**Figure 27:**
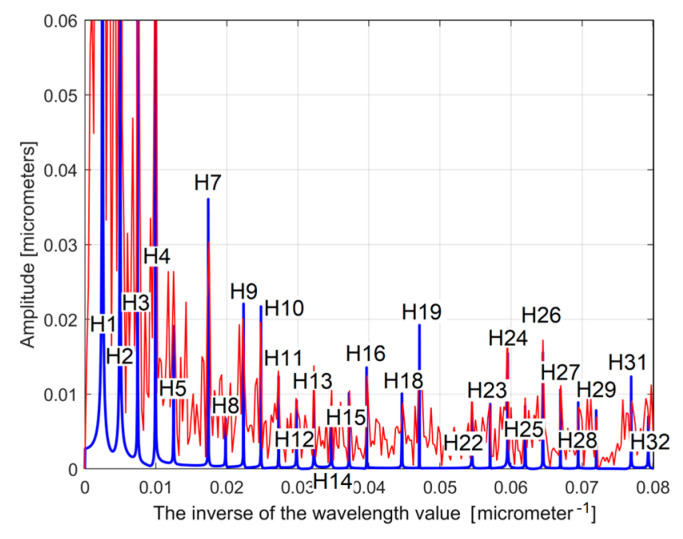
An extended view of the FFT spectra of the *y*(*x*) profile (in red) and the extrapolated *y_dhe_*(*x*) profile, with *p* = 10 (in blue). The peaks H1–H32 depict harmonic correlated components.

As a first example, related by the first 2DRP, Figure 26 shows partially (in the range 0 ÷ 0.02 μm^−1^ of conventional frequency) the FFT spectrum for the *y*(*x*) profile (in red, a spectrum already presented before in Figure 5)—and for the extrapolated *y_dhe_*(*x*) profile (in blue, with *p* = 10). Figure 27 presents both spectra over an extended conventional frequency range (0 ÷ 0.08 μm^−1^), with the first 27 harmonic correlated sinusoidal components (with *ω_Hi_*= *H_i_ ·ω_He_*_1_).

Because in this approach the conventional angular frequencies *ω_H_*_1_ and *ω_He_*_1_ have very similar values (Table 1 and Equation (5)), the FFT spectrum of the extrapolated *y_dh_*(*x*) and *y_dhe_*(*x*) profiles are very similar. Changing the origin of the *y_dhe_*(*x*) profile (to produce the *y_dhe_*_0_(*x*) profile) does not produce any change on the FFT spectrum (which is insensitive to the phase shifting). The FFT spectra of the extrapolated *y_dhe_*(*x*) and *y_dhe_*_0_(*x*) profiles are identical. 

**Figure 28:**
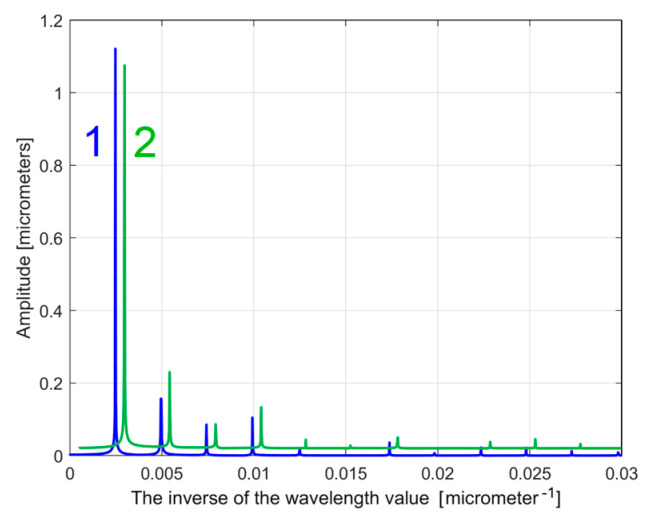
A partial view of the FFT spectra of extrapolated *y_dhe_*(*x*) profiles with *p* = 10; 1—for 1st 2DRP; 2—for 2nd 2DRP (shifted).

**Figure 29:**
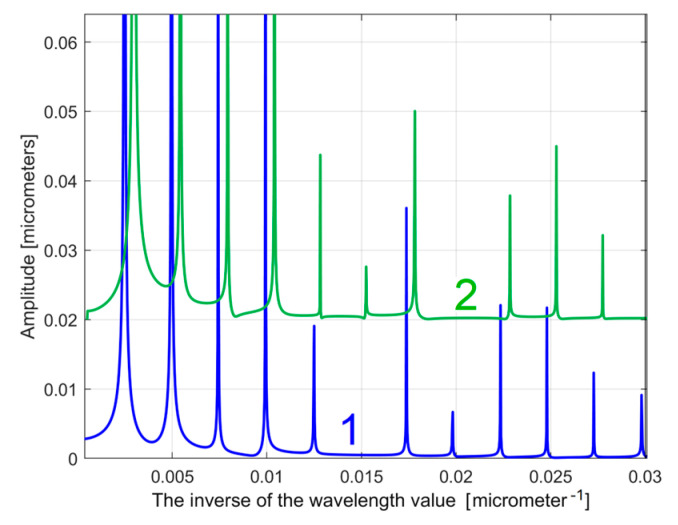
A zoomed image on *y*-axis of the FFT spectra from Figure 28.

It is obvious that the FFT spectrum of the extrapolated *y_dhe_*(*x*) profile can also be used as a pattern to compare two (or more) 2DRPs, sampled on the same surface, under identical conditions. The similarities between the partial FFT spectra of the extrapolated *y_dhe_*(*x*) profiles (with *p* = 10) found in both 2DRPs analysed before, are clearly highlighted in Figure 28, with a zoom on the *y*-axis shown in Figure 29. In both figures, in order to facilitate the comparison, the FFT spectrum of extrapolated *y_dhe_*(*x*) of the 2nd analysed 2DRP has been artificially shifted by 0. 02 μm upwards and 0.0005 μm^−1^ to the right.

A simpler and more reliable approach is to examine the resources provided by the compared FFT spectra of mathematically extended *y_ap_*_0_(*x*) patterns (related by both 2DRPs) over a large number of periods.

### 3.2. Analysis of 2D Roughness Profiles in the Feed Direction

A similar study can be made on the 2DRPs sampled on the same machined surface, in the feed direction, parallel to (d2), under identical conditions, number of samples, and sampling rate (sampling interval). Each of these 2DRPs is expected to describe a periodic succession of feed-interval scallops, as traces left by the tips of the milling tool edges during its rotation and feed motion. For a milling tool having three teeth, a 5600 rpm rotation speed, and a feed rate of 1560 mm/min, the conventional period of these feed-interval scallops should be equal to the feed per tooth *f_t_* = 0.1 mm. 

**Figure 30:**
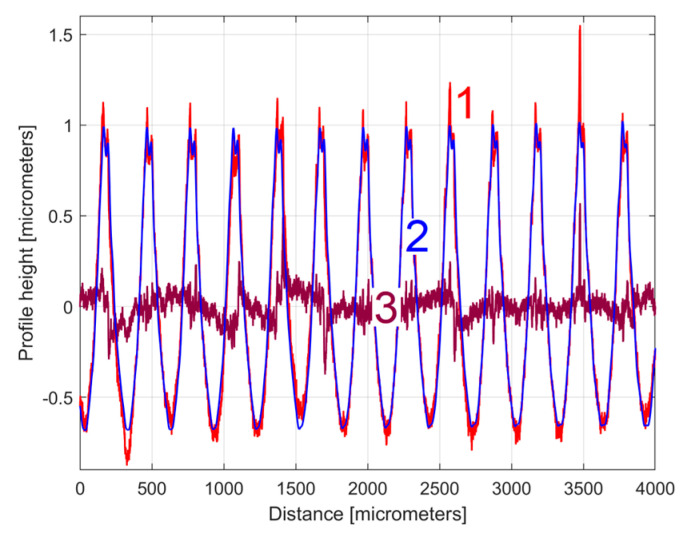
1—A first 2DRP; 2—The profile *y_dh_*(*x*) with 11 components; 3—The 11th residual *r*_11_(*x*).

**Figure 31:**
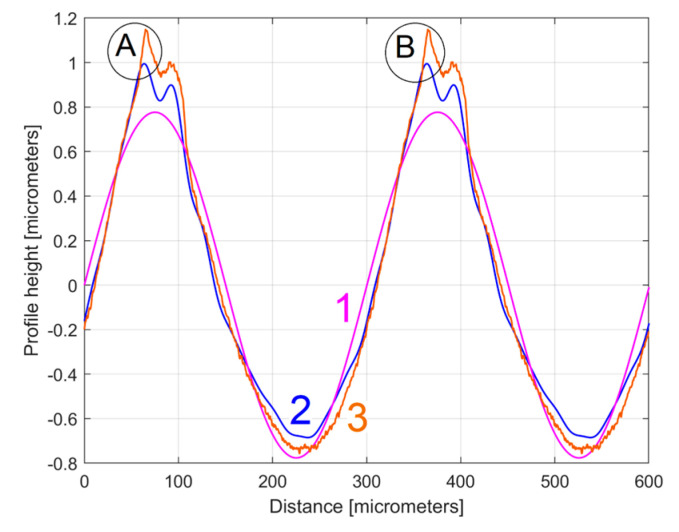
Some results of the analysis of the first 2DRP. Two conventional periods of: 1—the dominant component H1_0_; 2—the profile *y_dhe_*_0_(*x*); 3—the pattern *y_ap_*_0_(*x*).

Figure 30 shows a first 2DRP sampled in the feed direction (coloured in red), the deterministic harmonically correlated part *y_dh_*(*x*) (as a sum of 11 components, coloured in blue), and the residual *r*_11_(*x*) coloured in purple. Figure 31 shows the overlap of the first two periods of the dominant H1_0_ (curve 1), the first two periods of the profile *y_dhe_*_0_(*x*) (curve 2), and the pattern *y_ap_*_0_(*x*)—with *m* = 11—extrapolated on two periods (curve 3). As expected, there is a relatively good fit between them.

**Figure 32:**
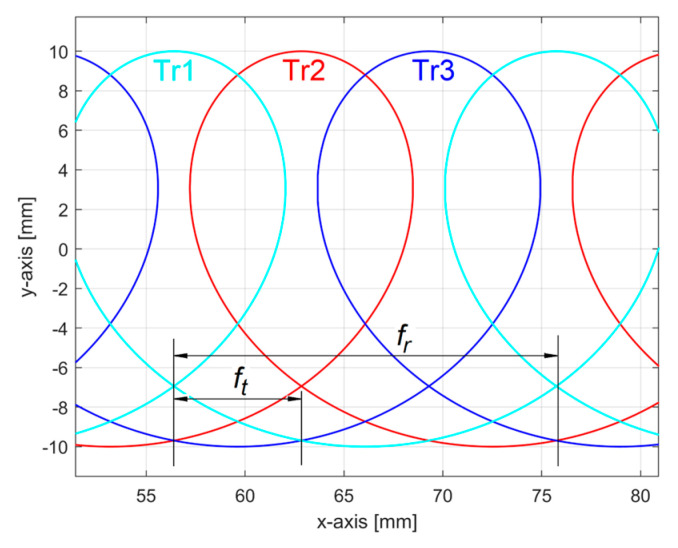
A simulation of 2D trajectories of points placed on teeth cutting edges (no run-out).

**Figure 33:**
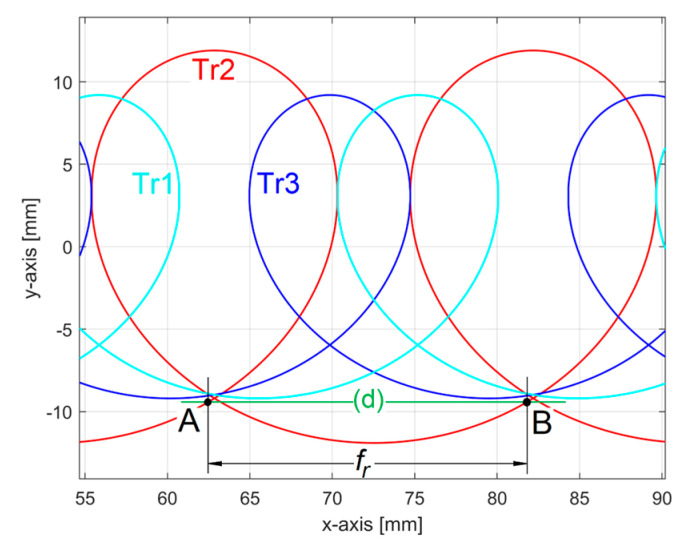
A simulation of 2D trajectories of points placed on teeth cutting edges (with run-out).

Unexpectedly, the conventional angular frequency *ω_He_*_1_ = 0.020921 rad/μm defines the wavelength λ*_He_*_1_ = 2*π*/*ω_He_*_1_ = 300.32 μm, as a conventional period, three times greater than the feed per tooth (100 μm), but practically equal to the feed per rotation *f_r_* of the milling tool. This means that the 2DRP in the feed direction reveals an abnormal behaviour of the milling tool, since because it turns off of its axis (with run out [3]), a single tooth is involved in the definition of the final machined surface (roughness). Obviously, the theoretical 2DRP in the feed direction consists mainly of a group of 2D curve (trochoidal) arcs, as parts of the trajectories of points on the teeth cutting edges. Figure 32 shows a conceptual simulation (without milling tool run-out) of these identical trochoidal trajectories (Tr1, Tr2, and Tr3) at a high feed rate (for clarity of approach). Figure 33 describes these trajectories with a particular run-out of milling tool: the centre of tool rotation is in opposite direction to the point involved in generating the trajectory Tr2. In both figures, for down milling, the theoretical 2DRP is described by arcs between the lowest intersection points of the trajectories.

If the run-out is large enough, then the theoretical 2DRP is described by arcs placed on a single trochoidal trajectory as in Tr2 in Figure 33. Here, (d) is the workpiece surface reference line before milling. A conventional period of the dominant component in 2DRP is equal with the feed per rotation (*f_r_*) and not with the feed per tooth (*f_t_* = *f_r_*/3). The points A, B in Figure 33 are located in the areas A, B in Figure 31. We should mention that, as opposed to Figure 33, Figure 31 does not have the same scale on the *x* and *y*-axis.

A similar and comparative study can be made in relation to a second 2DRP sampled on a straight line (feed direction) as a parallel direction to (d2) in Figure 1. As opposed to the analysis of the 2DRP in the pick direction, now this second 2DRP was sampled along a straight line carefully placed as accurately as possible over a whole number of pick intervals. A correct comparison requires that the first and second theoretical 2DRP should be the result of the trajectories of the same points on the teeth cutting edges.

**Figure 34:**
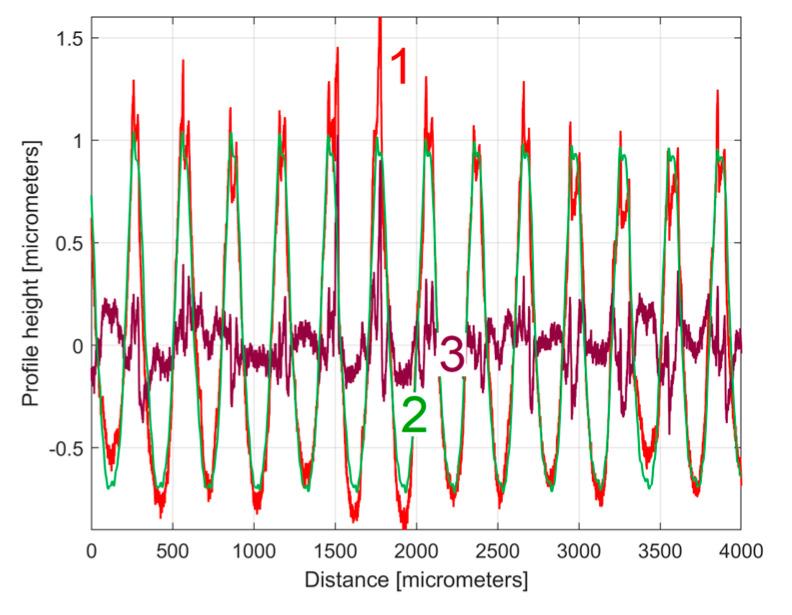
1—A second 2DRP; 2—The profile *y_dh_*(*x*) having 11 components; 3—The 11th residual *r*_11_(*x*).

**Figure 35:**
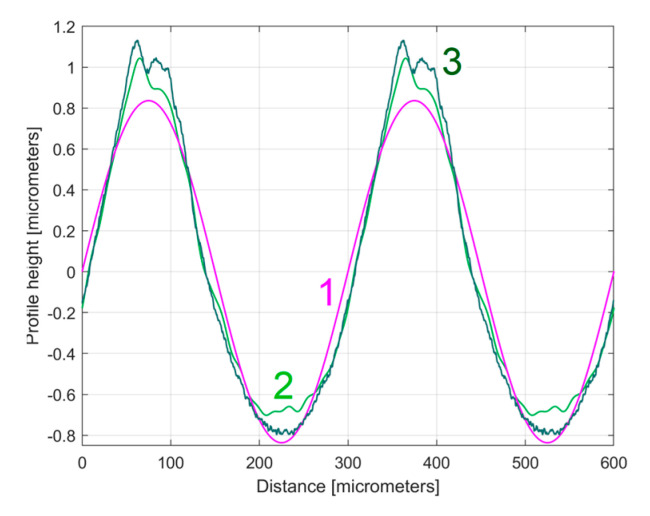
Some results of the analysis of the second 2DRP. Two conventional periods of: 1—the dominant component H1_0_; 2—the profile *y_dhe_*_0_(*x*); 3—the pattern *y_ap_*_0_(*x*).

The equivalent of Figure 30 is shown in Figure 34 and the equivalent of Figure 31 is depicted in Figure 35. As expected, similar to Figure 31, there is a relatively good fit between the dominant component H1_0_, the *y_dhe_*_0_(*x*) profile, and the pattern *y_ap_*_0_(*x*).

As previously stated, related by the first 2DRP in the feed direction, the same abnormal behaviour of the milling tool persists, because for the run-out it is the case that a single tooth is involved in defining the final machined surface, and the conventional angular frequency *ω_He_*_1_ = 0.020946 rad/μm defines the wavelength λ*_He_*_1_ = 2*π*/*ω_He_*_1_ = 299.97 μm, as a conventional period or feed per rotation *f_r_* (very close to that determined for the first profile), which is three times greater than the feed per tooth (100 μm).

**Figure 36:**
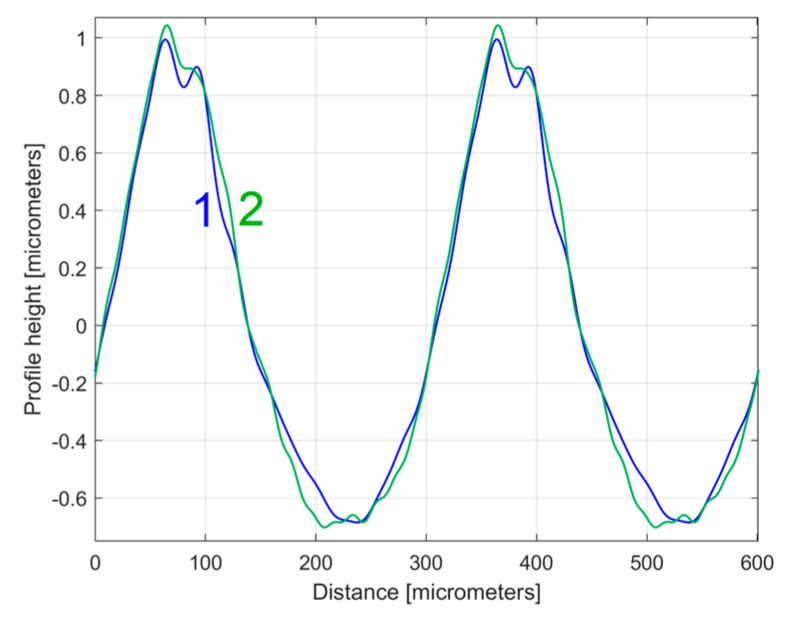
An overlap of the *y_dhe_*_0_(*x*) profiles (two periods) for the 1st and 2nd 2DRP (1 and 2).

**Figure 37:**
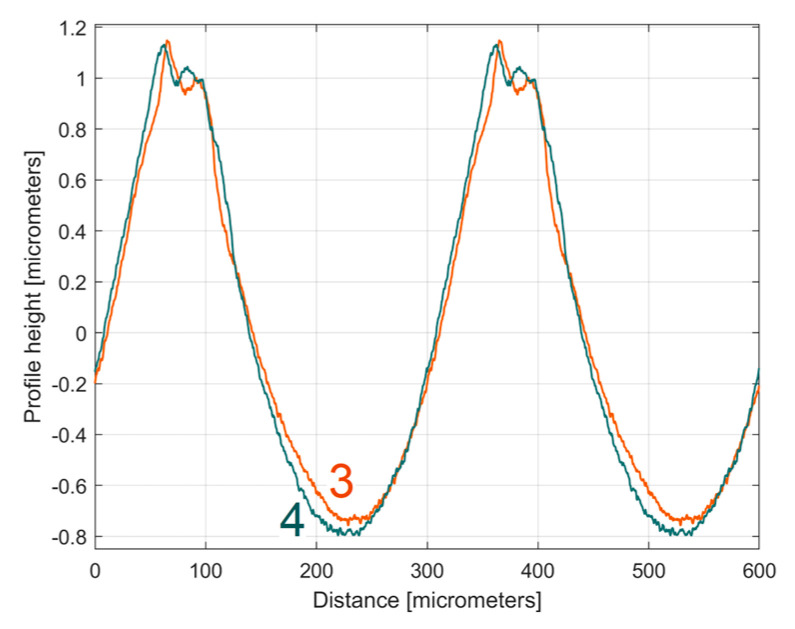
An overlap of the *y_ap_*_0_(*x*) patterns (two periods) for the 1st and 2nd 2DRP (3 and 4).

**Figure 38:**
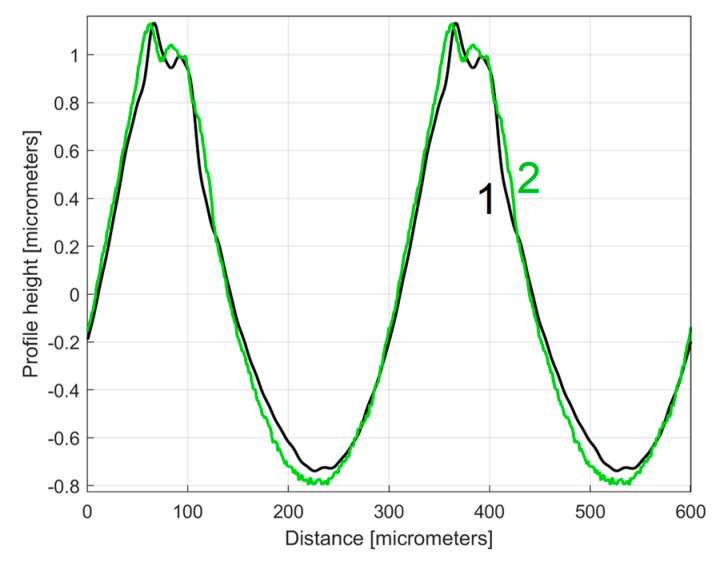
The first two periods of the *y_dhe_*_0*t*_(*x*) profiles: 1—for the 1st 2DRP; 2—for the 2nd 2DRP.

A comparison of Figure 31 and Figure 35 shows that, similar to the study in the pick direction, there are also strong similarities between these two different 2DRPs sampled in the feed direction. Figure 36 shows two periods of the overlapped profiles *y_dhe_*_0_(*x*), Figure 37 shows the overlap of the extended patterns *y_ap_*_0_(*x*) with two periods, and Figure 38 shows the first two overlapped periods of the *y_dhe_*_0*t*_(*x*) profiles.

However, it should be noted that the coincidence of these two *y_dhe_*_0_(*x*) profiles (Figure 36) is less good than in the case of the *y_dhe_*_0_(*x*) profiles for 2DRPs sampled in the pick direction (Figure 19). A similar conclusion can be drawn for the fit of the *y_ap_*_0_(*x*) patterns (by comparing Figure 23 and Figure 37) or for the *y_dhe_*_0*t*_(*x*) profiles (Figure 25 and Figure 38). The main reason for these mismatches is the lack of certainty that the two analysed 2DRPs were generated by the same points of the tool edges (an error that must be eliminated for an accurate analysis).

As already stated before, the description of the *y_dhe_*_0*t*_(*x*) profiles is more reliable (in relation to the *y_ap_*_0_(*x*) patterns) than the description of the *y_dhe_*_0_(*x*) profiles. 

**Figure 39:**
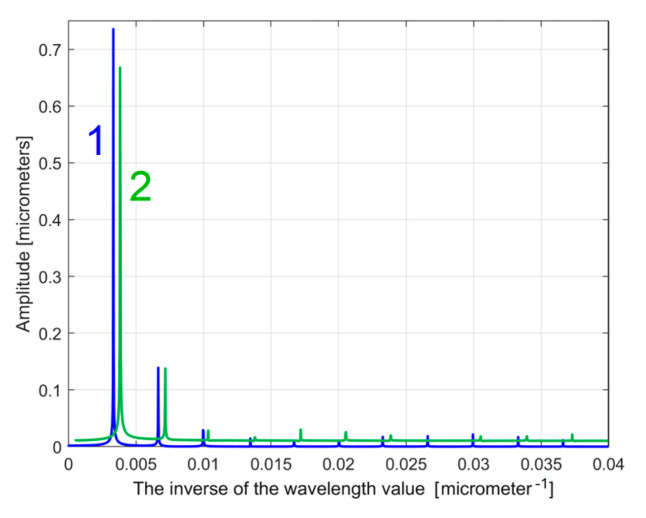
A partial view of the FFT spectra of the extrapolated *y_dhe_*(*x*) profiles with *p* = 10: 1—for the first 2DRP; 2—for the second 2DRP (shifted).

**Figure 40:**
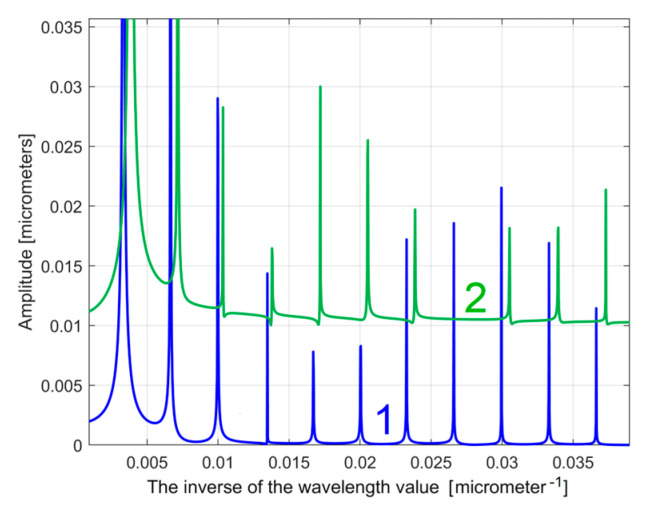
A zoomed image on the *y*-axis of the FFT spectra from Figure 39.

It is also possible to make a comparison between the FFT spectra of the extrapolated *y_dhe_*(*x*) profiles of both 2DRPs, with *p* = 10, as Figure 39 indicates, with zooming in on the *y*-axis, as shown in Figure 40. For easier comparison, the FFT spectrum of the extrapolated *y_dh_*(*x*) of the 2nd analysed 2DRP has been artificially shifted by 0.01 μm upwards and 0.0005 μm^−1^ to the right.

The similarities between spectra of the extrapolated *y_dhe_*(*x*) profiles are certainly related by conventional peak frequencies but less certainly related by the peak amplitudes. 

## 4. Conclusions

The proposed method for analysing and finding (by curve/signal fitting) the mathematical description of the periodic part of an experimental 2D roughness profile, 2DRP (as a sum of sinusoidal components harmonically correlated), provides reliable results, experimentally confirmed, useful for the characterisation of the milled surface (as a sum of wavinesses in two perpendicular directions), the interaction between the tool and workpiece during the milling process (in particular of flat surfaces machined with a ball nose end mill, constant step over), and the machinability of workpiece materials by a cutting process.

This paper proposes an analytical definition of a periodic profile as the best systematic characterisation (pattern) of an experimental 2DRP sampled with a contact profilometer (in pick and feed directions). A very similar periodic profile (but without an analytical description) is generated by a special type of sample averaging within the experimental 2DRP. These periodic profiles are useful for comparison purposes between different experimental 2DRPs, or to validate a predictive model for 2DRP [12,29,30], or to obtain the mathematical description of the microgeometry of a milled surface. 

As suggested during the review of this paper, a possible approach would be to use a curve fitting formula using the milling process parameters (and also tool condition and characteristics) as variables. This will be a challenge for a future approach. In the current approach (valid for any type of evolution of a physical quantity with a dominant periodic component), the fitting formula (a sinusoidal function), repetitively applied to obtain the best characterisation of the 2DRP profile as the sum of significantly harmonically correlated sinusoidal components (used as a pattern), indirectly provides some information related to the milling process, such as the peak to peak amplitude of the resultant is the pick-interval or feed-interval scallop height, and the conventional angular frequency of the fundamental describes the pick feed (pick direction 2DRP) or feed per tooth (feed direction 2DRP) as a relationship between tool rotation speed and feed rate. The analysis of the shapes of the experimental 2DRP patterns and the highlighting of differences with the theoretical patterns allows for the qualitative description of some anomalies of the machining process, such as the tool run-out (already shown in this paper), tool wear or cutting edges fracture, elastic bending deformation of the tool, etc.

This paper proves that the mathematical extrapolation of the analytically defined periodic profile of 2DRP improves the availability of a known but underutilized method of roughness analysis based on the spectrum of the periodic profile (seen as a time-dependent signal) generated by Fast Fourier Transform (FFT), with a low (conventional) frequency resolution.

Of course, generalisation of these results to the analysis of other types of milled surfaces, machined on other milling machines, with other types of tools on other workpiece materials (and possibly using other roughness sampling methods), is entirely feasible in a future approach.

As a future approach, we also intend to extend this study to the investigation of the 3D mathematical description of the roughness microgeometry of the complex milled surfaces, experimentally sampled with a suitable optical system.

## Figures and Tables

**Table 1:** The values of *A_Hi_*, *ω_Hi_*, and *φ_Hi_* involved in the mathematical description of 30 well harmonically correlated sinusoidal components within *y_dh_*(*x*) of the 2DRP, in the pick direction.

Harmonic # (Hi)	Amplitude *A_Hi_* [μm]	Conventional Angular Frequency *ω_Hi_* [rad/μm]	Wavelength *λ_Hi_* = 2π/*ω_i_* [μm]	Phase *φ_Hi_* at Origin (*x* = 0) [rad]
H1	*A_H_*_1_ = 1.148	*ω_H_*_1_ = 0.01557	*λ_H_*_1_ = 403.544	*φ_H_*_1_ = 4.2031
H2	0.2459	0.03118 (as 2.0025·*ω_H_*_1_)	201.53 (as *λ_H_*_1_/2.0024)	1.412
H3	0.09367	0.04669 (as 2.9987·*ω_H_*_1_)	134.57 (as *λ_H_*_1_/2.9988)	4.3261
H4	0.1116	0.06239 (as 4.0070·*ω_H_*_1_)	100.70 (as *λ_H_*_1_/4.0074)	2.456
H5	0.02461	0.07848 (as 5.0404·*ω_H_*_1_)	80.061 (as *λ_H_*_1_/5.0405)	3.085
H7	0.03816	0.1092 (as 7.0138·*ω_H_*_1_)	57.538 (as *λ_H_*_1_/7.0135)	4.785
H8	0.009202	0.1245 (as 7.9961·*ω_H_*_1_)	50.467 (as *λ_H_*_1_/7.9962)	5.8120
H9	0.0236	0.1404 (as 9.0173·*ω_H_*_1_)	44.752 (as *λ_H_*_1_/9.0173)	3.4731
H10	0.02267	0.1558 (as 10.0064·*ω_H_*_1_)	40.328 (as *λ_H_*_1_/10.0065)	4.6591
H11	0.0129	0.1714 (as 11.0083·*ω_H_*_1_)	36.658 (as *λ_H_*_1_/11.0083)	1.21
H12	0.01171	0.1873 (as 12.0295·*ω_H_*_1_)	33.546 (as *λ_H_*_1_/12.0296)	5.3275
H13	0.01317	0.2027 (as 13.0186·*ω_H_*_1_)	30.997 (as *λ_H_*_1_/13.0188)	1.796
H14	0.01174	0.2181 (as 14.0077·*ω_H_*_1_)	28.808 (as *λ_H_*_1_/14.0081)	5.8364
H15	0.01097	0.2337 (as 15.0096·*ω_H_*_1_)	26.885 as *λ_H_*_1_/15.0100)	5.3481
H16	0.01386	0.2493 (as 16.0016·*ω_H_*_1_)	25.203 (as *λ_H_*_1_/16.0117)	3.097
H18	0.01152	0.2805 (as 18.0154·*ω_H_*_1_)	22.399 (as *λ*_1_/18.0162)	5.5279
H19	0.0192	0.2961 (as 19.0173·*ω_H_*_1_)	21.219 (as *λ_H_*_1_/19.0180)	0.628
H22	0.01029	0.3425 (as 21.9974·*ω_H_*_1_)	18.345 (as *λ_H_*_1_/21.9975)	3.9651
H23	0.008842	0.3586 (as 23.0315·*ω_H_*_1_)	17.521 (as *λ_H_*_1_/23.0320)	2.8171
H24	0.02065	0.3741 (as 24.0270·*ω_H_*_1_)	16.795 (as *λ_H_*_1_/24.0276)	4.3321
H25	0.009471	0.3896 (as 25.0225·*ω_H_*_1_)	16.127 (as *λ_H_*_1_/25.0229)	1.299
H26	0.01693	0.4053 (as 26.0308·*ω_H_*_1_)	15.502 (as *λ_H_*_1_/26.0317)	1.822
H27	0.01081	0.4208 (as 27.0263·*ω_H_*_1_)	14.931 (as *λ_H_*_1_/27.0273)	4.2351
H28	0.009238	0.4365 (as 28.0347·*ω_H_*_1_)	14.394 (as *λ_H_*_1_/28.0356)	1.563
H29	0.007853	0.4524 (as 29.0559·*ω_H_*_1_)	13.888 (as *λ_H_*_1_/29.0570)	0.7781
H31	0.01326	0.4833 (as 31.0405·*ω_H_*_1_)	13.000 (as *λ_H_*_1_/31.0418)	0.5155
H32	0.01014	0.4985 (as 32.0167·*ω_H_*_1_	12.604 (as *λ_H_*_1_/32.0171)	3.9471
H38	0.008485	0.5924 (as 38.0475·*ω_H_*_1_)	10.606 (as *λ_H_*_1_/38.0487)	5.7423
H41	0.02497	0.6392 (as 41.0533·*ω_H_*_1_)	9.829 (as *λ_H_*_1_/41.0565)	1.478
H42	0.01782	0.6548 (as 42.0552·*ω_H_*_1_)	9.595 (as *λ_H_*_1_/42.0577)	0.9996

**Table 2:** The values *A_Hi_*, *ω_Hi_*, and *φ_Hi_* involved in the mathematical description of 12 harmonically correlated sinusoidal components within *y_dh_*(*x*) of the 2nd 2DRP, sampled in the pick direction.

Harmonic # (Hi)	Amplitude *A_Hi_* [μm]	Conventional Angular Frequency *ω_Hi_* [rad/μm]	Wavelength *λ_Hi_* = 2π/*ω_i_* [μm]	Phase *φ_Hi_* at Origin (*x* = 0) [rad]
H1	*A_H_*_1_ = 1.124	*ω_H_*_1_ = 0.01552	*λ_H_*_1_ = 404.8444	*φ_H_*_1_ = 0.4821
H2	0.2406	0.03099 (as 1.9968·*ω_H_*_1_)	202.7488 (as *λ_H_*_1_/1.9968)	0.407
H3	0.08159	0.04671 (as 3.0097·*ω_H_*_1_)	134.5148 (as *λ_H_*_1_/3.0097)	5.2621
H4	0.1232	0.06224 (as 4.0103·*ω_H_*_1_)	100.9509 (as *λ_H_*_1_/4.0103)	6.1897
H5	0.0234	0.07745 (as 4.9903·*ω_H_*_1_)	81.1257 (as *λ_H_*_1_/4.9903)	4.9971
H6	0.008268	0.09263 (as 5.9604·*ω_H_*_1_)	67.8310 (as *λ_H_*_1_/5.9684)	0.205
H7	0.03793	0.1088 (as 7.0103·*ω_H_*_1_)	57.7499 (as *λ_H_*_1_/7.0103)	3.7771
H9	0.01861	0.1404 (as 9.0464·*ω_H_*_1_)	44.7520 (as *λ_H_*_1_/9.0464)	0.1429
H10	0.02616	0.1558 (as 10.0387·*ω_H_*_1_)	40.3285 (as *λ_H_*_1_/10.0387)	3.6981
H11	0.01256	0.1712 (as 11.0309·*ω_H_*_1_)	36.7008 (as *λ_H_*_1_/11.0309)	2.608
H13	0.01106	0.2026 (as 13.0541·*ω_H_*_1_)	31.0128 (as *λ_H_*_1_/13.0541)	2.05
H22	0.0107	0.3425 (as 22.0683·*ω_H_*_1_)	18.3451 (as *λ_H_*_1_/22.0683)	1.326

## Data Availability

The data presented in this paper are available upon request addressed to the corresponding author. The data are not publicly available due to privacy.
